# Single- and Double-Comb Tilted Fibre Bragg Grating Refractive Index Demodulation Methods with Fourier Transform Pre-Processing

**DOI:** 10.3390/s22062344

**Published:** 2022-03-18

**Authors:** Sławomir Cięszczyk, Krzysztof Skorupski, Patryk Panas

**Affiliations:** Institute of Electronics and Information Technology, Lublin University of Technology, 20-618 Lublin, Poland; k.skorupski@pollub.pl (K.S.); p.panas@pollub.pl (P.P.)

**Keywords:** TFBG sensors, double comb, refractive index, Fourier transform

## Abstract

The development of fibre optic sensors for measuring the refractive index is related to the creation of new periodic structures and demodulation algorithms for the measured spectrum. Recently, we proposed a double-comb Tilted fibre Bragg grating (DCTFBG) structure. In this article, we analyse such a structure for measuring the refractive index in comparison to a single classical structure. Increasing the number of modes causes a significant change in the Fourier spectrum of optical spectra. For the purpose of data pre-processing, we propose the Fourier Transform as a filtering method in the frequency domain. Then, we analyse separately the band-filtered optical spectra for several frequency ranges. For quantitative analysis, we use algorithms that use quantitative changes in the transmission, i.e., the method of the envelope and the length of the spectrum contour. We propose the use of the Hilbert transform as the envelope method. The second type of algorithms used are methods determining the shift of spectrum features along the wavelength axis. The method of determining the centre of gravity of the area bounded by the envelope and the maximum of the second derivative of the smoothed cumulative spectrum contour length is proposed here. Using the developed methods, the measurement resolution was achieved at the level of 2 × 10^−5^ refractive index unit.

## 1. Introduction

Periodic fibre structures are used as sensors of various quantities. Tilted fibre Bragg gratings (TFBG) are structures with a characteristic transmission spectrum containing several dozen resonances creating a dense comb of cladding modes. Such gratings are sensitive to temperature [1], stress [2], bending [3] and external refractive index [4,5]. Their main application is the measurement of the surrounding refractive index (SRI) which is associated with a significant modification of the cladding mode comb. Since the tilted grating introduces asymmetry, the cladding modes are also sensitive to light polarisation. As a result of the tilting of the periodic structure in the fibre core, some of the power from the core is transferred to the cladding. Modes created in this way are sensitive to changes of environment surrounding the cladding. Each of the modes has a characteristic value of the effective refractive index. If the SRI exceeds this value, the mode becomes a leaked mode. This results in the disappearance of such resonance in the spectrum of the grating. The cut-off wavelength indicates the transition between the guided mode and the leakage of the cladding. The mode at the cut-off point is very sensitive to even slight changes in SRI. The changes in the spectrum of tilted gratings take place in such a way that an indirect mapping using transmission changes or projecting its changes on the wavelength axis can be used to determine the information contained therein.

Tilted fibre Bragg gratings are an element of various measuring systems. In order to improve the metrological parameters of sensors with TFBG, various modifications of such structures are proposed. For example, cascade connections of two structures are used. One of such systems is a combination of two TFBG written on a typical single-mode fibre (SMF) and SMF with reduced diameter [6]. The effect of such a combination is the creation of two spectra combs shifted to each other so that there is a concentration of resonance dips in the range of cladding modes. The reduction of the separation between cladding modes can also be obtained for a complex structure stored in the same place in the fibre. A double-comb TFBG (DCTFBG) allows for the reduction of the separation between peaks for a specific range of cladding modes [7]. This structure is made up of two gratings written on the same section of the optical fibre. In the inscription process, first one and then another grating is produced. Both gratings slightly differ in terms of their period, which causes a mutual shift in their spectra. This shift is chosen to be half the distance between the resonances of a single grating. Another type of modification of the tilted structure is tilted Moire fibre Bragg gratings. They are used in the case of simultaneous measurement of two parameters (temperature and SRI) with a single grating [8]. Cascade gratings connecting several gratings with different tilt angles allow for the measurement of a very wide range of SRI [9]. Additional sensitivity and selectivity are obtained by surface plasmon resonance-tilted fibre Bragg gratings. Such properties are obtained by appropriately covering the optical fibre with thin metallic layers. The new properties of the gratings can be obtained using the line-by-line inscription method [10].

**Figure 1:**
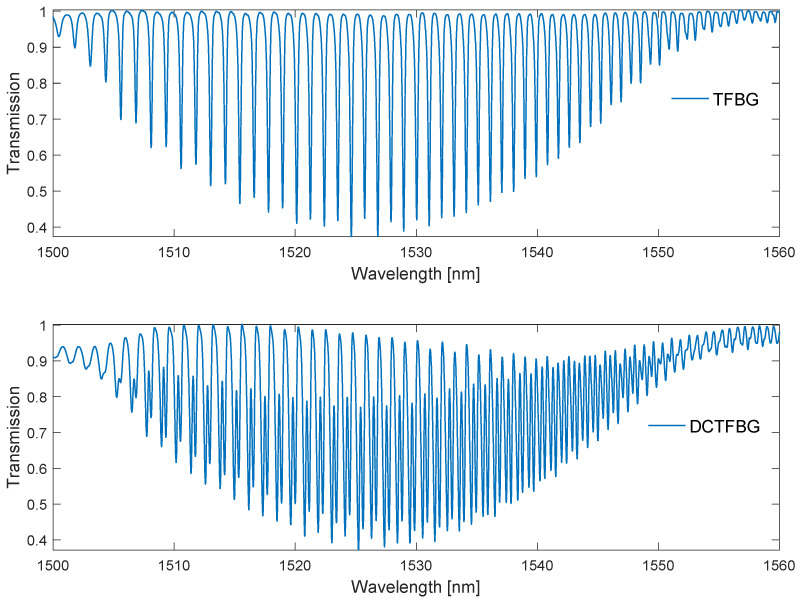
Spectrum of a TFBG and of a DC-TFBG, the latter being one of the gratings produced by us.

The basic parameters of tilted gratings are the tilt angle Θ, grating period Λ and effective refractive index of the fibre core of the grating. Physical parameters of the optical fibre itself, i.e., the diameter of the core and the cladding, are no less important. Reducing the diameter of the optical fibre causes a greater intensity of resonance peaks and increases the influence of the surrounding environment on the transmission spectrum [11]. The smaller diameter increases the distance between the individual cladding modes [12]. By controlling the manufacturing process, specific spectral characteristics of the gratings can be achieved. This allows for the achievement of the planned SRI measurement range and the necessary measurement sensitivity. Using advanced technological operations, it is possible to create a structure with a doubled number of cladding modes [7], whose structure will be analysed later in the article. Figure 1 shows a comparison of the spectra of a single and double grating. In the DCTFBG, the adjacent modes are visible over the entire spectral range. In the 1505–1525 nm range, the additional resonances are close to the original ones. In the range of 1530–1548 nm, the distances between the individual modes are even. DCTFBG may have an even distribution of modes in different parts of the spectrum. For further analysis, a specially produced grating was used, in which the uniform distribution of modes occurs in the range of lower and middle wavelengths.

Increasing the number of modes can be advantageous in two main ways. In the case of a very precise calibration of the SRI value corresponding to the cut-off wavelength of each individual mode, it is possible to reduce the stepped nature of the sensor processing characteristic. This is the result of increasing the number of points of such a characteristic [13,14]. The second way to use the increase in the number of cladding modes is the case of direct use of the most popular algorithm for calculating the area of the cladding mode region. Doubling the number of mode peaks allows a simpler and more accurate way of estimating the area of the cladding modes.

In order to determine the value of the refractive index using a measuring system with a TFBG, it is necessary to calibrate such a sensor and a spectral demodulation method. Spectrum demodulation algorithms can be divided into those using the amplitude changes of the transmission spectrum and using the wavelength shift of specific spectral parameters. A single mode, a group of modes and the entire spectral range of cladding modes can be analysed. Detailed analysis and review of demodulation methods will be presented in the next chapter.

The aim of this article is to determine the properties of DCTFBG in the case of measuring the refractive index. At the same time, we are introducing a new approach to spectrum demodulation algorithms, proposing new algorithms and modifications to existing algorithms. Due to significant differences in the Fourier spectra for TFBG and DC-TFBG, we propose the use of filtration of the measured optical spectra in the Fourier domain. The optical spectrum can be analysed separately in individual frequency bands. Generally known demodulation algorithms can be applied to such filtered data. Additionally, we propose modification of popular algorithms by calculating the envelope using the Hilbert transform. Next, we calculate the parameters related to the determination of shifting of the cladding mode changes by calculating the centre of gravity of the envelope area as well as the maximum wavelength of the second derivative of the smoothed cumulative spectral contour length.

Later in the article, methods of analysing the spectra of TFBG will be presented, and then their modifications will be proposed. The differences in their optical and Fourier spectra for TFBG and DC-TFBG will be presented successively. Then, for the newly created gratings, the spectrum demodulation algorithms for individual band filtering will be compared.

## 2. Methods of TFBG Spectra Analysis

Many different methods of TFBG spectrum analysis have been proposed in the literature. The first proposed method was to determine the SRI dependency on the basis of cladding mode envelopes. In the original version, the method was based on determining the maxima and minima of the cladding mode comb to determine the normalised area occupied by the cladding mode [15]. The more precise version uses the Delaunay triangulation method [16,17]. These methods belong to the group that uses information about changes in the transmission coefficient for the entire range of the cladding modes. A variation of this type of method is the integrated differential area method [18]. It calculates the value of the absolute difference between the spectrum for the analysed SRI and the reference spectrum, i.e., for pure water. The integrated transmission value for the specified spectral range of the modes is then calculated. This range is selected depending on the measuring range for the SRI. The methods using the differences between the reference spectrum and the measured spectra are also used in spectral interrogation for plasmon resonance (SPR) sensors [19]. It was also proposed to calculate the correlation between the reference spectrum envelopes and the individual measured spectra. From such correlation product, statistical parameters such as Skewness and Kurtosis [20] are determined.

The methods using information about changes in the amplitude of the cladding modes for the entire transmission spectrum may also be based on the differences in the spectrum amplitudes in relation to its local average value. The first proposed algorithm of this type is an algorithm that calculates the value of the standard deviation between the spectrum amplitudes and the local mean of the spectrum [21]. Another parameter of this type is the determination of the average difference from the local average [22]. The spectrum contour length parameter can also be calculated [23]:

(1) $$L=\sum_{i=0}^{N-1}\left|T_{i+1}-T_{i}\right|,$$

where *T_i_* are measured spectrum points in the specified spectral range.

The second group of methods uses changes in transmission parameters (amplitude and wavelength) for a single mode. However, these methods have limited applicability for the smaller SRI ranges. They are also characterised by significant non-linearity. Since the shift of the cladding modes and the Bragg mode is equally sensitive to temperature, when determining the SRI based on the spectral shift of one mode, the Bragg mode shift is additionally used as a temperature indicator [24]. Generally, the higher order modes are more sensitive to changes in SRI; however, their scope of application is smaller.

For very small SRI changes, significant for some biosensors, the difference spectrum for one mode can be used [25]. Additionally, in order to better visualise a single mode, this method uses additional changes in the polarisation of light.

The last group of methods uses the entire range of the cladding mode spectrum to determine the cut-off wavelength. This is the place on the wavelength axis where the cladding modes become leakage modes. This position changes with the SRI value. Interesting results are obtained if the TFBG spectrum is filtered in the Fourier transform domain before determining the cut-off wavelength [26]. After removing the constant component and components with higher frequencies, the spectrum becomes symmetrical with respect to the wavelength axis. The cut-off wavelength is determined by looking for the site on the wavelength at which the filtered cladding mode amplitudes begin to decrease. In order to avoid a jump between individual modes, the authors propose an approximation with a smooth arc tangent curve between the peaks of the filtered spectrum at its top and bottom. The cut-off wavelength is the place where the second derivative is zero.

Another method of spectrum demodulation uses an algorithm consisting of three steps [27]. In the first one, the spectrum is smoothed, which will be used as a reference spectrum. Then, the difference between the currently studied spectrum and the reference spectrum is determined on a decibel scale. Then, for the entire calibration set, the place of intersection of the differential spectrum with the horizontal reference line on the shorter wavelength side is determined. The authors developed an algorithm for the optimal selection of the reference threshold level. This intersection point, of course, shifts towards longer wavelengths as the SRI increases.

In the [22] article, it is proposed to use the curve created as a result of calculating the cumulative version of locally calculated parameters such as cumulative mean deviation from the local mean:

(2) $$CMD(k)=\frac{1}{k}\cdot \sum_{i=1}^{k}\left|T_{i}-{\bar{T}}_{i}\right|.$$

where ${\bar{T}}_{i}$ are spectrum points smoothed by the lowpass filter.

Due to the different nature of changes in SPR-TFBG spectra, the methods of their analysis are modifications of the methods developed for TFBG or they are dedicated methods. However, when synthesising a new algorithm, it is also worth getting acquainted with them, because they can indicate a new approach or modification of already existing methods. One of the more interesting methods of information extraction from SPR-TFBG spectra is to use the Fourier spectrum for this using the FFT transform [28]. Another interesting method is the determination of the location of the constriction in the transmission spectrum [29]. The spectrum of the grating is filtered in the domain of the Fourier transform.

## 3. Frequency Analysis of Optical Spectra of TFBG

**Figure 2:**
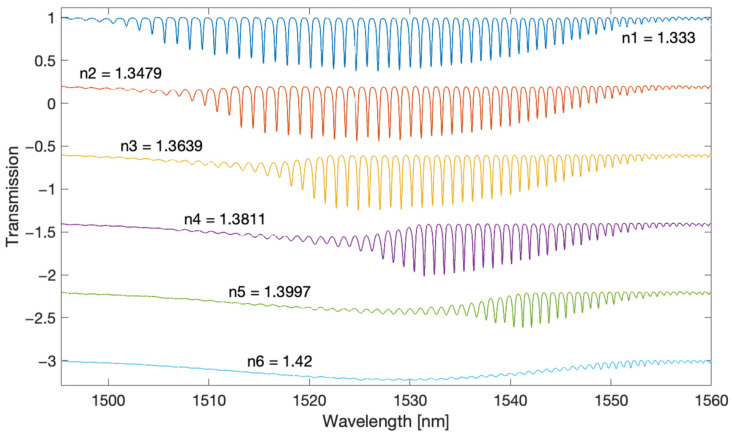
Optical spectrum of the TFBG for several SRI values.

**Figure 3:**
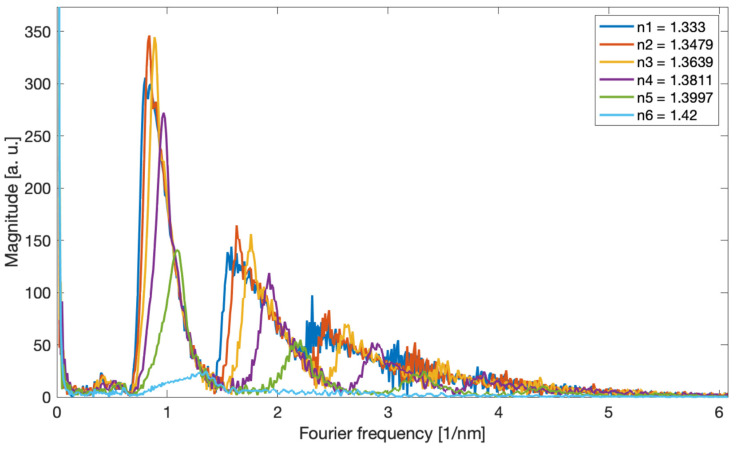
The amplitude Fourier spectrum corresponding to the optical spectra of the TFBG in Figure 2, depending on the SRI.

For the measurements, we used tilted gratings with a tilt angle of 6 degrees, made by us with the use of an excimer laser (Coherent Inc., Santa Clara, CA, USA) using the phase mask method. The radiation source was a super-luminescent S5FC1005S diode (Thorlabs Inc., Newton, NJ, USA). Optical spectra were measured with the optical spectrum analyser AQ6370D (Yokogawa, Tokyo, Japan) with a resolution of 0.02 nm. The measured SRI ranged from 1.333 to 1.42. Figure 2 shows the optical spectra of a single TFBG grating which is immersed successively in glucose solutions of increasing refractive index value. Figure 3 shows the amplitude frequency spectra calculated using the Fourier transform. The optical spectrum of the grating has cladding mode peaks that are unevenly distributed. The greatest distances between the modes are for shorter wavelengths. As the wavelength increases, the distance between the modes decreases. The optical spectrum can be treated as a signal with the frequency content changing along with the wavelength. In the Fourier spectrum, frequencies from 0.6 to 1.4 1/nm can be defined as a frequency band associated with the occurrence of the cladding modes. Such a signal is a kind of chirp type signal. From the frequency 1.4 to 2.2 1/nm, it is possible to observe the next harmonic, which can be described as the second band signal.

The Fourier transform allows the frequency analysis of the measured spectra and their filtering by using the inverse transform. The fundamental frequency in the TFBG spectrum is related to the cladding mode resonances, which are distributed unevenly in the transmission spectrum. The greatest distances in the spectrum are from the side of the shorter wavelengths and therefore it corresponds to the lowest frequency in the Fourier spectrum. For cladding modes closer to the Bragg and Ghost modes, the distances between the modes are smaller, which corresponds to a higher Fourier frequency. Increasing the SRI causes the smallest frequencies to disappear. The parameter that represents the change in the frequency content of the spectrum, and thus indirectly also the SRI, is the centroid frequency [29].

**Figure 4:**
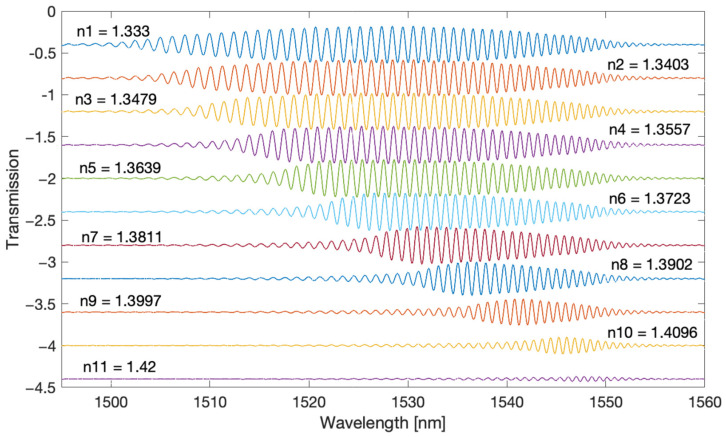
TFBG spectra after Fourier filtering for the first (main) frequency band.

**Figure 5:**
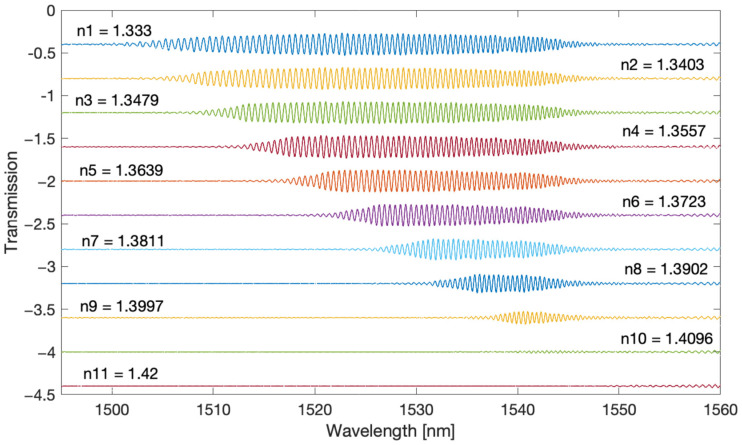
TFBG spectra after Fourier filtering for the second frequency band.

**Figure 6:**
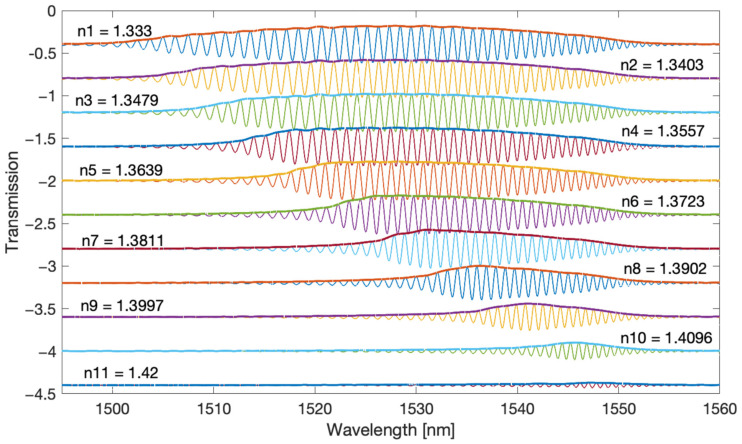
Envelope calculated by the Hilbert transform method for the main frequency band.

**Figure 7:**
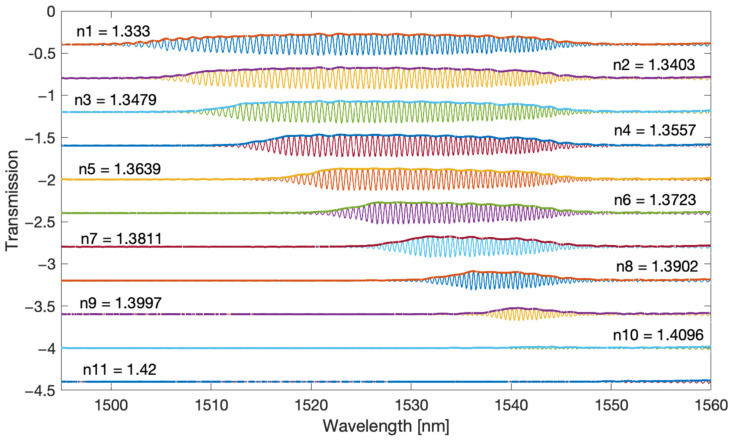
Envelope calculated by the Hilbert transform method for the second frequency band.

The filtered spectra shown in Figure 4 and Figure 5 are chirp signals, i.e., they contain a sinusoidal signal with a frequency increasing with the wavelength and changing amplitude. Figure 6 and Figure 7 present the envelopes calculated by the Hilbert transform method for the first and second frequency bands. With this method, the envelope can only be determined for sinusoidal signals. It is therefore not possible for the original optical spectrum of the TFBG. The popular methods of calculating the envelope applied so far use only information from the maxima and minima of the optical spectrum. In the case of the Hilbert transform, the envelope is computed taking into account all spectral points.

In the case of spectrometric interrogation of TFBG, the metrological parameters of a given measurement method depend on two main factors: the properties of the entire measurement path and the method of determining a specific parameter on the basis of which the SRI is calculated. The mathematical method used may be resistant to unwanted changes in the spectrum, such as noise or baseline fluctuations related to changes in the radiation source. Fourier filtering seems to be the optimal method for pre-processing optical spectra.

## 4. Comparison of Demodulation Algorithms for TFBG and DCTFBG 

**Figure 8:**
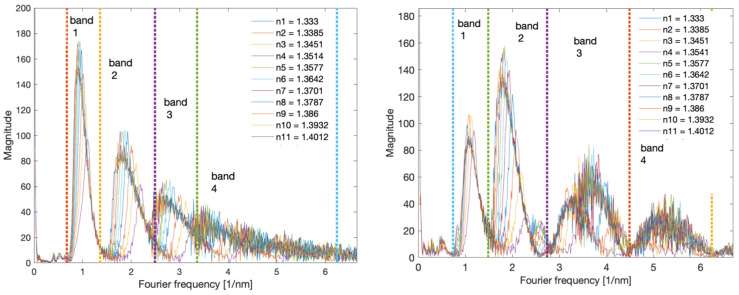
Comparison of Fourier spectra of TFBG and DC-TFB gratings for individual SRIs.

Figure 8 shows the Fourier spectra of the prepared TFBG and DCTFBG. The gratings were immersed in a glucose solution with SRI values from 1.333 to 1.4012. On the left side it is a classic TFBG grating, while on the right side there is a grating with an increased number of modes. The frequencies from 0.7 to 1.5 1/nm contain the fundamental frequencies related to the shape of the cladding mode group. The next frequency band from 1.5 to 2.5 1/nm is associated with twice as many frequent features of the optical spectrum. Therefore, for the DCTFBG grating, the amplitude of the doubled frequency is 60% higher than the fundamental frequency, while for the TFBG grating, the amplitude of the second frequency is 40% lower. Likewise, the higher amplitudes contain the frequencies associated with the third band in the spectrum. However, this third band for the classical grating is between 2.5 and 3.5 1/nm, while for the DC-TFBG grating, the third frequency band is between 3 and 4.5 1/nm. Subsequent increase of SRI causes shifting of spectra in particular distinguished bands. This is due to the disappearance of the cladding modes at lower wavelengths. Thus, the smaller Fourier frequencies disappear because for smaller wavelengths, the distances between the modes are smaller.

The purpose of creating new gratings for testing algorithms was to achieve a similar depth (transmission) for both types of gratings. The second goal is to increase the number of modes in the leftmost part of the grating spectrum, i.e., where the distances between the modes are the greatest. Since it is this range of the optical spectrum that also seems to be the most frequently used for SRI measurements, dealing with a part of it is important.

**Figure 9:**
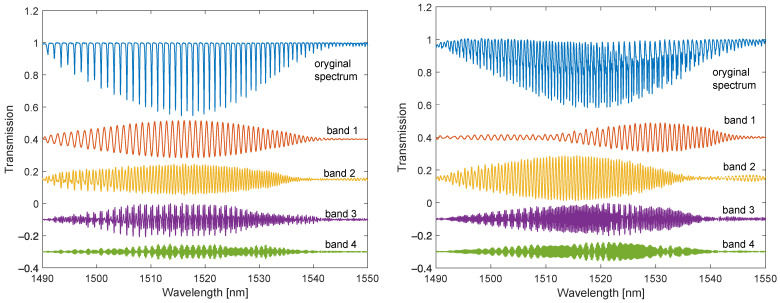
Optical spectra: original and band-filtered for individual frequency ranges for the TFBG and DC-TFBG (SRI = 1.333).

In Figure 9 we can see the basic optical spectra measured for both gratings immersed in distilled water. The spectra of the individual bands of the Fourier spectra are presented below the actual optical spectra. For the classic TFBG, the first component contains the highest amplitude. Its amplitude changes smoothly over the entire width of the optical spectrum (1490–1540 nm). For the DC-TFBG, the first frequency has a significant amplitude for a wavelength greater than 1515 nm. The second frequency band for the TFBG has a similar envelope to the first band. For the DC-TFBG, the second frequency envelope is significantly larger, in the range from 1490 to 1530 nm. A decrease in the value of the second frequency envelope from 1520 nm is simultaneously observed with an increase in the envelope of the first frequency band.

**Figure 10:**
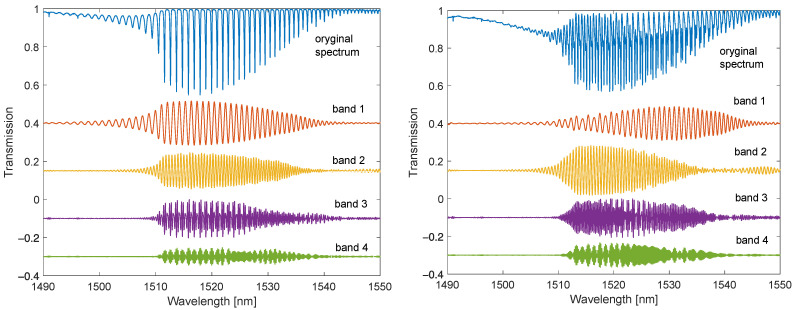
Optical spectra: original and filtered for individual frequency bands for TFBG and DC-TFBG (SRI = 1.3701).

**Figure 11:**
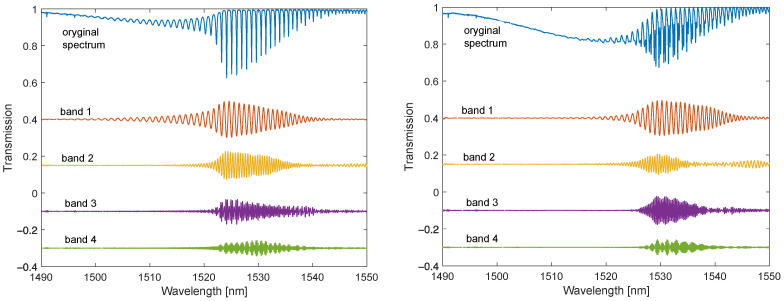
Optical spectra: original and band-filtered for individual frequency bands for TFBG and DC-TFBG (SRI = 1.4012).

In Figure 10 and Figure 11, one can observe the disappearance of the modes from the shorter wavelength side with the increasing SRI. The individual envelopes for the original spectrum and the spectra of the individual Fourier bands disappear. For SRI = 1.37, the fading is below 1512 nm, and for SRI = 1.4, it is below 1525 nm. For the DC-TFBG, a lower transmission value is visible in the part of the spectrum where the modes have leaked. This is the result of the greater power delivered to the optical fibre during the DCTFBG recording process. This increases the average value of the refractive index in the optical fibre core.

The Fourier spectra presented so far and the Fourier filtered optical spectra can be treated as signals for quantitative analysis for the determination of the SRI coefficient. Fourier transform filtering can then be considered as a pre-processing step of the signals. Each of the filtered signals can be subjected to algorithms determining the appropriate parameter, which will represent the SRI coefficient using the calibration curve.

**Figure 12:**
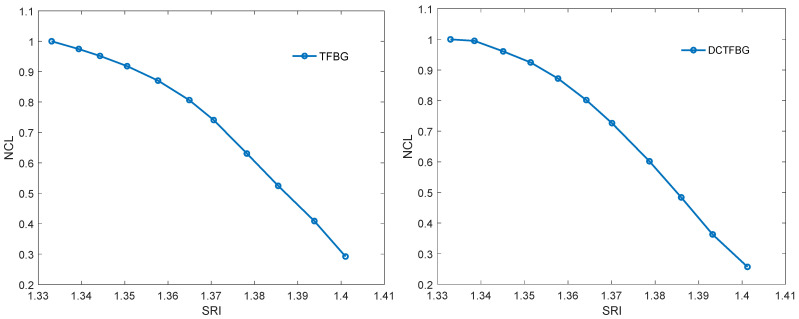
Change of the normalised contour length from SRI for the TFBG and DC-TFBG.

The first algorithm used is that to calculate the spectrum length. It is unambiguously the simplest algorithm, because it only requires the calculation of the sum of the absolute differences of individual spectrum elements. The characteristics of the normalised spectral length as a function of the refractive index are shown in Figure 12. Table 1 compares the resolutions obtained with multiple measurements. For each SRI value, the measurement was repeated 20 times. The comparison shows that the resolution obtained for both types of gratings is comparable. A clearly worse result is the resolution for the DC-TFBG and the band for the first frequency range. This is due to the fact that the content of the first band is significantly reduced for the DC-TFBG and the amplitude of this signal is small for shorter wavelengths. This is the effect of the increased number of cladding modes described earlier in the discussion of Figure 8 and Figure 9. 

**Figure 13:**
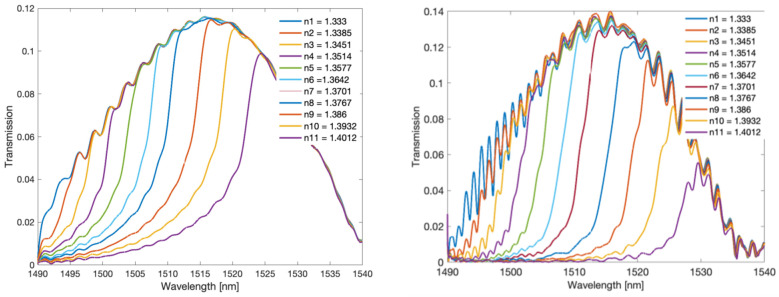
Changing the spectrum of the Hilbert envelope from SRI for band 1 of the TFBG and band 2 for the TCTFBG.

**Figure 14:**
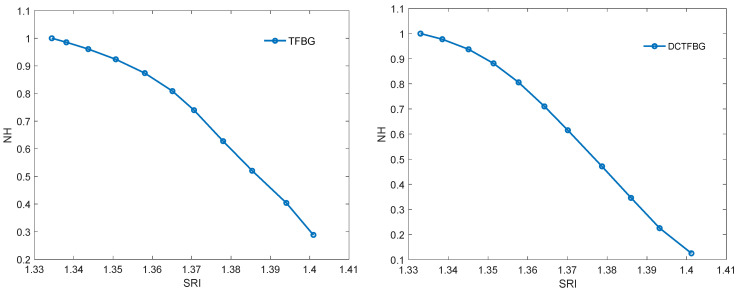
Change of normalised Hilbert envelope from SRI for TFBG (band 1) and DCTFBG (band 2).

Another algorithm is the envelope algorithm. It is conceptually the first algorithm proposed for mode decay analysis for TFBG. The original algorithms, however, consisted in determining the envelope by searching for peaks in the transmission spectrum. The peaks (maxima) for the upper envelope and the minima for the lower envelope were searched separately. More advanced algorithms such as the Hilbert transform can also be used to calculate the envelope. However, it will not affect the basic unfiltered TGBG spectrum because it is a set of many frequencies. The Hilbert transform itself enables the calculation of the envelope but for sinusoidal signals. Since the filtered signal for each frequency band approximately satisfies this assumption, the Hilbert transform can be used. Filtered signals are symmetrical about the amplitude axis, hence there is no need to calculate the upper and lower envelopes. The shape of the Hilbert envelope for the spectra for individual SRIs is shown in Figure 13. For the TFBG grating, the envelopes for the first frequency band are presented because it has the greatest amplitude. For the DC-TFBG, the envelope for the second frequency band is shown as it is the most representative band for this structure. The dependence of the normalised area bounded by the Hilbert envelope is shown in Figure 14. Table 2 compares the resolution calculated on the basis of multiple measurements for the area determined by the Hilbert envelope method. As for the previous algorithms, we get a worse result for the first frequency band for the DC-TFBG.

**Figure 15:**
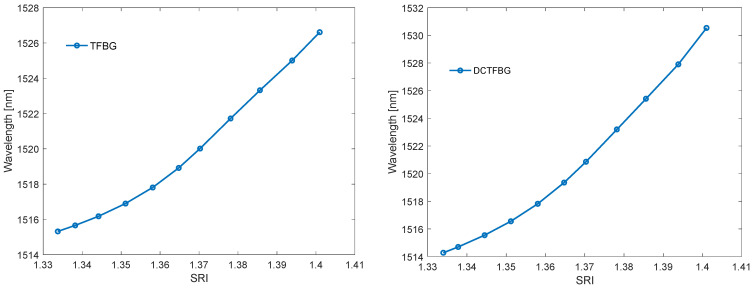
Change in the wavelength of the centre of gravity of the TFBG optical spectrum after band filtration for band 1 and for DCTFBG for band 2 filtration.

The algorithms presented so far calculated a certain amplitude parameter of the filtered spectrum. The fading out of the cladding modes reduces both the area delimited by the envelope and the length of the spectrum. This fading is related to the shift of the cut-off wavelength towards the longer wavelengths. Figure 13 shows that the centre of gravity may be such an indicator of the displacement of the envelope and the surface area. The change of the wavelength of the centre of gravity is shown in Figure 15. Table 3 shows the resolutions of the proposed method. The resolutions of the centre of gravity method do not differ from the previous amplitude methods; however, a better linearity of the calibration characteristics should be noted (Figure 15).

**Figure 16:**
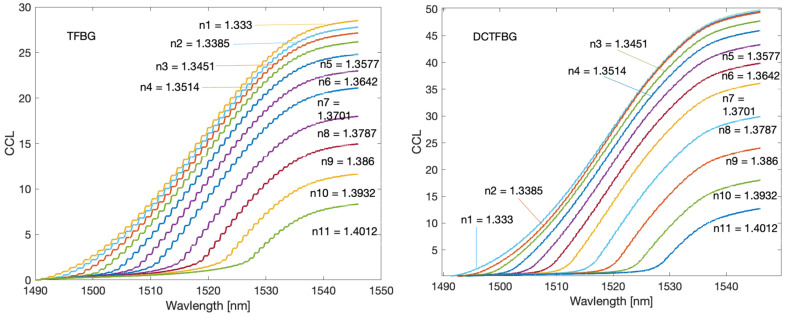
Cumulative spectrum contour for TFBG and DC-TFBG.

**Figure 17:**
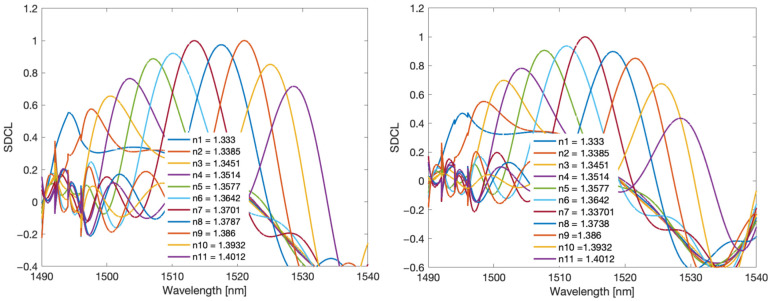
Second derivative of the cumulative spectrum contour for TFBG and DCTFBG.

**Figure 18:**
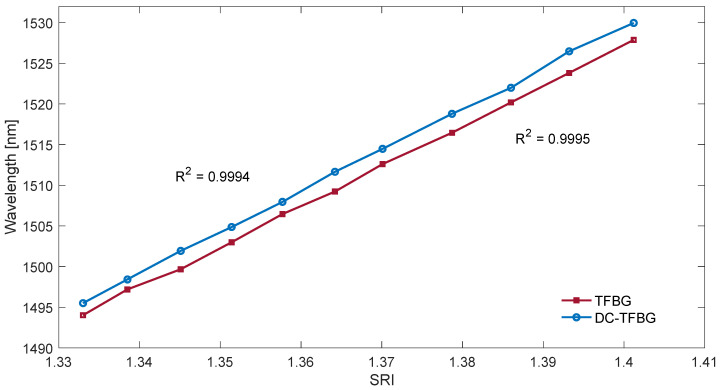
The wavelength of the second derivative maximum of the cumulative contour spectrum for TFBG and DCTFBG.

Cumulative spectral length plots are shown in Figure 16. The calculations were performed for the original unfiltered optical spectra. The noticeable difference between the two types of gratings here is the more pronounced stepped nature of the cumulative curves for the TFBG. As the calculated quantities have not been normalised, the greater length of the spectrum for DC-TFBG is clear, resulting from a greater number of modes. The cumulative spectral length curves evidently shift with increasing SRI. This shift can also be projected onto the wavelength axis. In a physical sense, the cut-off wavelength can be determined at the point where the cumulative curves begin to increase significantly. It is possible to find this place using the second derivative. It will have the maximum value for the place where the basic curve has the greatest increment. The derivative calculations require prior smoothing of the basic curves. Any low pass filter that will eliminate the stepped nature of the cumulative spectral length can be used here. The frequency spectrum shown in Figure 8 can be used to select the filter. In this case, the stop band of the designed low-pass filter should contain the first chirp band if we analyse the basic unfiltered spectrum of both types of gratings. Universal smoothing filters such as a moving average or Savitzky–Golay can also be used. The maximum value of the second derivative of the optical spectrum shown in Figure 17 shifts linearly towards longer wavelengths with increasing SRI as can be seen in Figure 18. Table 4 compares the resolution of the SRI measurement by searching for the maximum of the second derivative of cumulative spectral length. In terms of resolution of multiple measurements, this method is the best method presented in this article.

## 5. Conclusions

The article compares two types of structures: the classic TFGB and a new type of grating DCTFBG with an increased number of modes. The comparison was made in the field of the Fourier transform. The proposed new approach is the division of the frequency spectrum into several bands and a separate analysis of the filtered optical spectrum. Both the basic optical spectrum and each of the four bands in question were analysed using two types of algorithms. The amplitude of optical spectrum change algorithms was the spectrum length and the area calculated on the basis of the Hilbert envelope. The algorithms that use projection on the wavelength axis include change of the centre of gravity of the area bounded by the envelope and the wavelength of maximum value of the second derivative of the cumulative spectral length.

The basic conclusion from the performed measurements and calculations is that the Fourier domain filtering results in no significant differences in the resolution of the demodulation algorithms. This is a confirmation of the well-known fact that properly performed data pre-processing significantly improves the parameters of the entire quantitative data analysis process. From the practical point of view, after properly performed Fourier filtration, the simplest demodulation methods can be used. However, it should also be noted that the best resolution (2 × 10^−5^ refractive index unit) is provided by methods of projection onto the wavelength axis, especially the second derivative of the cumulative spectral length.

## Figures and Tables

**Table 1:** Comparison of the resolution for the TFBG and DC-TFBG for optical spectra for individual frequency ranges.

	TFBG	DC-TFBG
Contour length, original spectrum	7 × 10^−5^	6.2 × 10^−5^
Contour length, band 1	4.3 × 10^−5^	3.7 × 10^−4^
Contour length, band 2	4.29 × 10^−5^	6.5 × 10^−5^
Contour length, band 3	7.55 × 10^−5^	8.0 × 10^−5^
Contour length, band 4	1.5 × 10^−4^	1.25 × 10^−4^

**Table 2:** Comparison of the resolution for the Hilbert envelope method for the TFBG and DC-TFBG for the optical spectra for individual frequency ranges.

	TFBG	DC-TFBG
Hilbert envelope, band 1	4.0 × 10^−5^	3.8 × 10^−4^
Hilbert envelope, band 2	3.9 × 10^−5^	5.9 × 10^−5^
Hilbert envelope, band 3	6.5 × 10^−5^	7.9 × 10^−5^
Hilbert envelope, band 4	1.53 × 10^−4^	1.4 × 10^−5^

**Table 3:** Comparison of the resolution for the Hilbert envelope centre of gravity method for the TFBG and the DC-TFBG for optical spectra for individual frequency ranges.

	TFBG	DC-TFBG
Center of gravity, band 1	6.5 × 10^−5^	2.8 × 10^−4^
Center of gravity, band 2	9.6 × 10^−5^	6.7 × 10^−5^
Center of gravity, band 3	9.0 × 10^−5^	5.8 × 10^−5^
Center of gravity, band 4	1.56 × 10^−4^	1.8 × 10^−4^

**Table 4:** Comparison of the resolution for wavelength of the maximum of the second derivative for the TFBG and the DCTFBG for optical spectra for individual frequency ranges.

	TFBG	DC-TFBG
Second derivative, whole spectrum	2.5 × 10^−5^	2.01 × 10^−5^
Second derivative, band 1	1.9 × 10^−5^	4.5 × 10^−4^
Second derivative, band 2	1.8 × 10^−5^	2.4 × 10^−5^
Second derivative, band 3	3.0 × 10^−5^	1.8 × 10^−5^
Second derivative, band 4	2.9 × 10^−4^	4 × 10^−5^

## Data Availability

Not applicable.
